# Supplementary material for: Tumorigenicity decrease in Bcl-xL deficient MDCK cells ensuring the safety for influenza vaccine production
Source: PLoS One. 2024 Dec 16;19(12):e0311069. doi: 10.1371/journal.pone.0311069 (PMC11649150; doi:10.1371/journal.pone.0311069)
Supplement: S1 File — (ZIP) [file pone.0311069.s002.zip › S2_File/4_KOG_enrichment/network/B_vs_A.sign_KOG_enrichment_network.pdf]

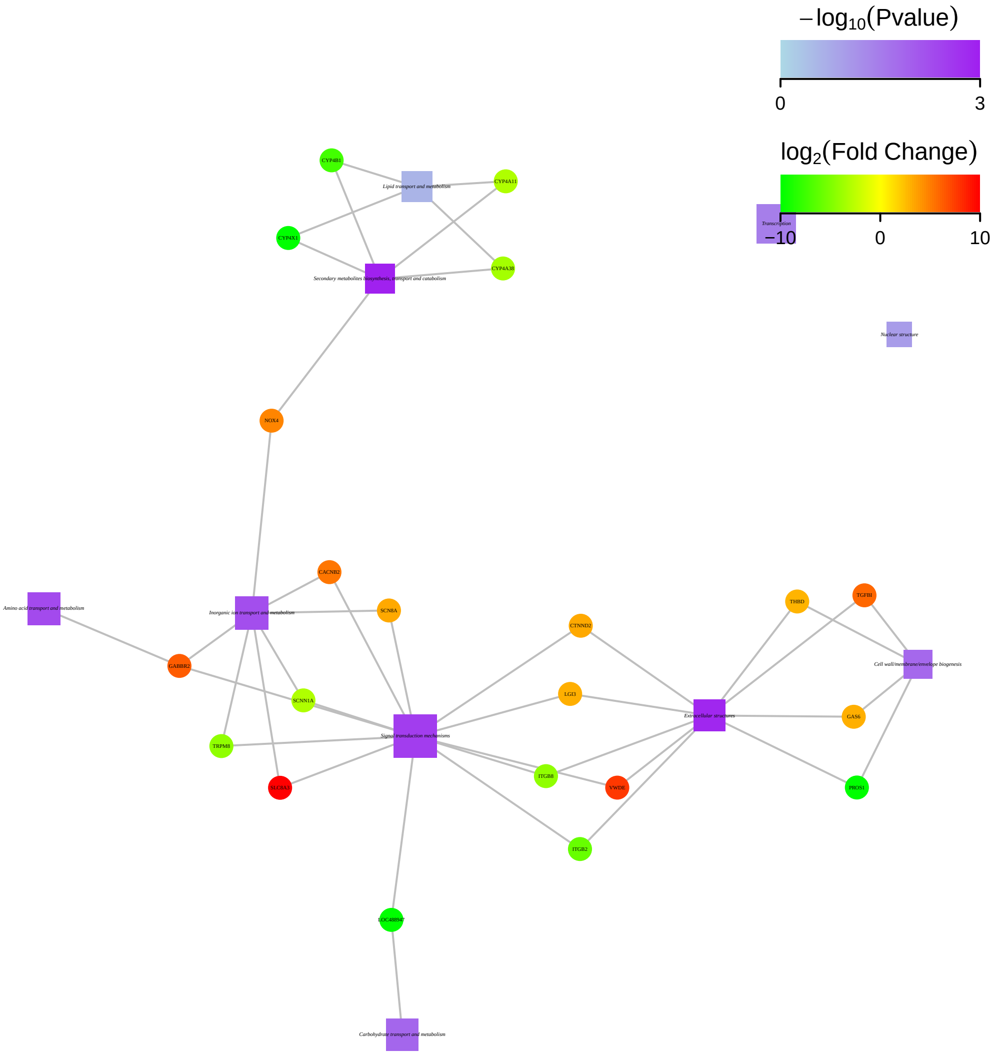

$-\log_{10}(Pvalue)$
0
3
$\log_2(Fold\ Change)$
−10
0
10
Transcription
Nuclear structure
Lipid transport and metabolism
Secondary metabolites biosynthesis, transport and catabolism
CYP4B1
CYP4A11
CYP4X1
CYP4A38
NOX4
Amino acid transport and metabolism
Inorganic ion transport and metabolism
CACNB2
SCN8A
GABBR2
SCNN1A
TRPM8
SLC6A1
Signal transduction mechanisms
CTNND2
LGI3
ITGB8
VWDE
ITGB2
Extracellular structures
THBD
TGFBI
Cell wall/membrane/envelope biogenesis
GAS6
PROS1
LOC488947
Carbohydrate transport and metabolism
